# Exposure to Household Air Pollution from Biomass Cookstoves and Levels of Fractional Exhaled Nitric Oxide (FeNO) among Honduran Women

**DOI:** 10.3390/ijerph15112544

**Published:** 2018-11-13

**Authors:** Megan L. Benka-Coker, Maggie L. Clark, Sarah Rajkumar, Bonnie N. Young, Annette M. Bachand, John R. Balmes, Robert Brook, Tracy L. Nelson, John Volckens, Steve J. Reynolds, Ander Wilson, Christian L’Orange, Nicholas Good, Casey Quinn, Kirsten Koehler, Sebastian Africano, Anibal Osorto Pinel, Jennifer L. Peel

**Affiliations:** 1 Department of Environmental and Radiological Health Sciences, Colorado State University, Fort Collins, CO 80523, USA; maggie.clark@colostate.edu (M.L.C.); sarah.rajkumar@yahoo.de (S.R.); bonnie.young@colostate.edu (B.N.Y.); abachand@ramboll.com (A.M.B.); john.volckens@colostate.edu (J.V.); stephen.reynolds@colostate.edu (S.J.R.); n.good@colostate.edu (N.G.); jennifer.peel@colostate.edu (J.L.P.); 2 Department of Health Sciences, Gettysburg College, Gettysburg, PA 17325, USA; 3 Division of Occupational and Environmental Medicine, University of California, San Francisco, San Francisco, CA 94143, USA; john.balmes@ucsf.edu; 4 Division of Cardiovascular Medicine, University of Michigan Medical School, Ann Arbor, MI 48109, USA; robdbrok@med.umich.edu; 5 Department of Health and Exercise Science, Colorado State University, Fort Collins, CO 80523, USA; tracy.nelson@colostate.edu; 6 Department of Mechanical Engineering, Colorado State University, Fort Collins, CO 80523, USA; christian.lorange@colostate.edu (C.L.); casey.quinn@colostate.edu (C.Q.); 7 Department of Statistics, Colorado State University, Fort Collins, CO 80523, USA; ander.wilson@colostate.edu; 8 Department of Environmental Health and Engineering, Johns Hopkins University Bloomberg School of Public Health, Baltimore, MD 21205, USA; kkoehle1@jhu.edu; 9 Trees, Water & People, Fort Collins, CO 80524, USA; sebastian@treeswaterpeople.org (S.A.); anibalosorto@yahoo.es (A.O.P.); 10 Asociación Hondureña para el Desarrollo, Tegucigalpa, MDC, Honduras

**Keywords:** fractional exhaled nitric oxide, household air pollution, particulate matter

## Abstract

Household air pollution is estimated to be responsible for nearly three million premature deaths annually. Measuring fractional exhaled nitric oxide (FeNO) may improve the limited understanding of the association of household air pollution and airway inflammation. We evaluated the cross-sectional association of FeNO with exposure to household air pollution (24-h average kitchen and personal fine particulate matter and black carbon; stove type) among 139 women in rural Honduras using traditional stoves or cleaner-burning *Justa* stoves. We additionally evaluated interaction by age. Results were generally consistent with a null association; we did not observe a consistent pattern for interaction by age. Evidence from ambient and household air pollution regarding FeNO is inconsistent, and may be attributable to differing study populations, exposures, and FeNO measurement procedures (e.g., the flow rate used to measure FeNO).

## 1. Introduction

Exposure to household air pollution from the combustion of solid fuels was estimated to be responsible for 2.5 million deaths and 77.2 million DALYS (disability-adjusted life years) in 2016 [1]. The incomplete combustion of solid fuels used for cooking and heating, such as wood or crop residue, emits extremely high levels of particulate matter (PM) and black carbon [2,3], among other pollutants [4]. The resulting household air pollution is associated with adverse health outcomes including chronic obstructive pulmonary disease (COPD), lung cancer, adult lower respiratory infections, cardiovascular disease [5,6] and adverse pregnancy outcomes [7]. Household air pollution is a modifiable exposure, and cleaner-burning cookstove technologies have demonstrated the potential to increase cooking efficiency and reduce human exposure to PM_2.5_ (fine particulate matter) by 50% or more [8].

The underlying mechanisms of pulmonary diseases associated with air pollution are not well understood; however, evidence suggests that exposure may result in increased reactive oxygen species and production of proinflammatory cytokines, leading to airway inflammation [9,10,11,12,13]. Previous household air pollution studies have focused on COPD, acute lower respiratory diseases, and forced expiratory volume; however, other pulmonary impacts, such as asthma and airway inflammation, have not been well studied [5].

Fractional exhaled nitric oxide (FeNO) measured in human breath is a non-invasive method to assess subclinical airway inflammation [14]. A measure of FeNO quantifies nitric oxide (NO) as an indication of eosinophilic inflammation initialized by cytokines and Type 2 helper cells (Th2) [15,16,17,18] and may provide unique information on airway inflammation to complement spirometry [19]. Acute and chronic exposure to air pollution may result in airway inflammation, which can potentially be quantified by measuring FeNO. Several previous studies have demonstrated that ambient air pollution exposure [20,21,22,23] and wood smoke exposure [24] are associated with increased levels of FeNO among healthy adults; other studies have observed null finding with ambient air pollution or woodsmoke and FeNO [25]. Strong evidence exists for association between exposure to household air pollution and respiratory disease such as COPD and lung cancer [26,27], while evidence for an association with asthma is inconclusive [28]. To our knowledge, only one study has assessed the association between household air pollution and FeNO, reporting a small increase in FeNO (2 ppb) immediately after cooking [29]. This study was limited in that it evaluated the association between household air pollution and FeNO using a measure of “stove type” as the exposure. Directly monitoring both kitchen and personal exposure to household air pollution for a 24-h period will improve models evaluating the association.

Although there is some evidence supporting the association of air pollution and increased FeNO among healthy adults, little is known about the association of household air pollution and FeNO. Measures of FeNO may provide insight into airway inflammation as a result of household air pollution studies. Our objective was to evaluate the cross-sectional association of exposure to household air pollution (measured personal concentrations and kitchen concentrations of air pollutants and stove type) with exhaled nitric oxide in adult women in rural Honduras using traditional and cleaner-burning *Justa* stoves; we additionally evaluated the effect modifying role of age on the observed relationship.

## 2. Materials and Methods

All study protocols were approved by the Colorado State Institutional Review Board. All study participants provided informed consent and received food items worth $5 U.S. dollars.

### 2.1. Study Setting

**Figure 1:**
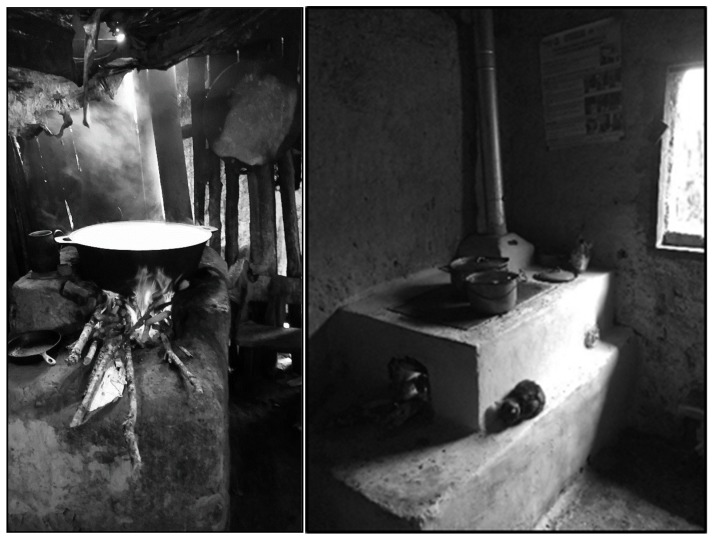
Typical traditional (**left**) and *Justa* (**right**) cookstoves in the homes of Honduran women.

The study was conducted in nine communities surrounding the town of La Esperanza, Honduras. La Esperanza, located in the mountainous region of Western Honduras, is home to approximately 15,000 people. Our target population included all female primary cooks who used a traditional cookstove or a cleaner-burning *Justa* cookstove (See Figure 1). Traditional cookstoves in our population are typically self-built wood-burning stoves, with a metal griddle, large combustion chamber, and possibly a chimney. The cleaner-burning *Justa* stove is a common wood-burning cleaner-burning stove in Latin America with a rocket-elbow combustion chamber, chimney, and metal griddle suited to making tortillas.

### 2.2. Participants

The study team held local community meetings in villages surrounding La Esperanza and presented detailed information regarding the study to the community members. Researchers screened 500 households that attended the community meetings and expressed interest in the research project. We utilized convenience sampling to locate households who owned cleaner-burning *Justa* stoves, and identified nearby households that owned a traditional stove. In total, we visited 170 households from 9 February–30 April 2015. We recruited the female primary cook in each household to participate in the study. Enrollment criteria required that the primary cook be age 25–56, a non-smoker, not pregnant, and must have owned a traditional cookstove or *Justa* cookstove since at least 4 months prior to the interview. Upon visitation, eighteen of the 170 households were excluded as they did not have a female primary who meet the eligibility criteria. Two women chose not to participate in the study. We enrolled a total of 150 women into the study.

### 2.3. Exposure to Household Air Pollution

**Figure 2:**
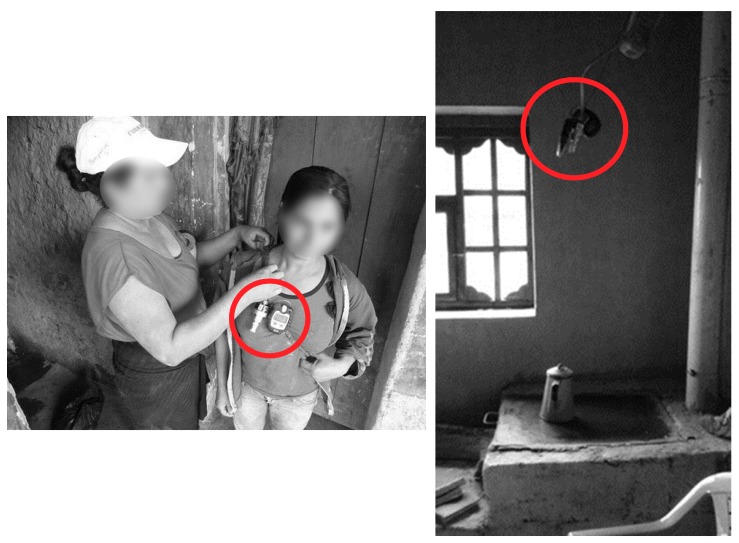
Example placement for kitchen exposure measurements (**left**) and personal measurements (**right**).

We assessed exposure to household air pollution using stove type (traditional or *Justa*) and by measuring 24-h average personal concentrations and kitchen concentrations of PM_2.5_ and black carbon. For kitchen concentrations, monitors for PM_2.5_ were placed in the kitchen between 76 and 127 cm above the stove, in the area where a woman cooks, and away from open windows and doors. For personal concentration measurements, we placed PM_2.5_ monitors in a small bag that each woman wore throughout the monitoring period, except when bathing or sleeping. Women were asked to place the bag next to them (on a table or hanging on the wall) when not wearing it. The inlet was clipped to the shoulder strap at the front of the woman’s chest near her breathing zone (Figure 2).

PM_2.5_ was collected on 37-mm PTFE-coated glass fiber filters (Fiberfilm^TM^ T60A20, Pall Corporation, Port Washington, NY, USA). The filters were equilibrated at controlled temperature and relative humidity for at least 24-h and then pre-weighed at Colorado State University (CSU) using the microbalance (Mettler Toledo Microbalance, model MX5, resolution and repeatability of 1-ug). In the field laboratory, filters were placed into Triplex cyclones with a particle cut size of 2.5 µm (BGI by Mesa Labs, Butler, NJ, USA). Cyclones were attached to pumps (SKC AirCheck XR5000, SCKInc, Eighty Four, PA, USA) with a flow rate of 1.5 L/min. Pumps were pre-calibrated daily using a flow meter (DryCal Dc-Lite, Bios International, Mesa Labs, NJ, USA). We collected one filter blank every two weeks. After collection of the sample, filters were stored at −22 °C and then transported to CSU, equilibrated for temperature and relative humidity, and post-weighed. The 24-h average PM_2.5_ concentration was calculated from the change in filter mass adjusted for the average blank mass. We calculated the limit of detection (LOD) for PM_2.5_ as follows: average mass of blanks +3 times the standard deviation of the sample masses. All samples with a concentration less than the LOD (7 kitchen samples and 7 personal samples) were replaced with a value of LOD/√2. Due to a broken DryCal pump needed to calibrate the equipment used for PM_2.5_, we were unable to gather PM exposure measures from 41 houses. In addition, other samples were excluded from analysis due to; AirCheck pumps ran for less than 75% of the intended time (<18 h) (three personal and two kitchen samples); faulty post-weight (one personal sample); missing post-calibration data in the field (one kitchen sample).

Black carbon concentrations were estimated based on the optical transmission of light through the air sampling filters [30] using a transmissometer (model OT-21, Magee Scientific, Berkeley, CA, USA). Transmission data were converted to mass concentrations based on published mass-absorption values for combustion aerosol [31] and corrected for a filter loading artefact that leads to an underestimation of the estimation at high sample loading [32]. The limit of detection was estimated to be 0.86 µg/m^3^, which corresponds to three times the standard deviation of 54 blank samples (additional blank filters were used from field sampling campaigns conducted within the same year to estimate the reference values for the transmissometer since pre-sampling transmission data were not collected on sample filters). Values below the LOD (kitchen: *n* = 3; personal: *n* = 10) were substituted by LOD/√2. Detailed information on black carbon is available as supplementary materials.

### 2.4. Fractional Exhaled Nitric Oxide

FeNO was measured at a flow rate of 50 mL/s within a range of 5 to 300 ppb using a NIOX MINO (Version 9, Aerocrine AB, Solna, Sweden). Each participant completed the FeNO measurement on the morning after the 24-h exposure assessment. Participants stood upright, emptied their lungs, inhaled steadily through the NIOX MINO, and then exhaled at a slow and steady rate for 10 s. Two participants had a value for the FeNO below the limit of detection (LOD) of 5 ppb; we replaced these values with the limit of detection divided by the √2. There were no values above 300 ppb. Additional health outcomes, including blood pressure and diabetes, were evaluated in our study population and published elsewhere [33,34].

### 2.5. Covariate Assessment

Questionnaires in Spanish were administered to obtain demographic data and anthropometric information in the homes of participants. Responses were entered into the electronic data collection system, Open Data Kit [35]. As indicators of socioeconomic status, we measured the number of beds per person in the household, years of formal education, electricity availability, number of assets (cars, bikes, motorbikes, televisions, radios, refrigerators, sewing machines, electricity), and a dietary-diversity score for each participant. To determine an individual’s dietary-diversity score, women were asked to report all food they had eaten in the previous 24-h period including the number of portions [36]. The final dietary-diversity score was a sum of the number of food groups a woman had eaten at least one portion of in the past 24 h, ranging from 1 to 10. Elevation of the household was measured using Maps.me, a cell-phone GPS application (My.com B.V., Version 6.5.3).

The body mass index (BMI) (kg/m^2^) and waist-to-hip ratio was measured for each woman as measures of obesity. Women self-reported symptoms of nose irritation, cough, mucus, and difficulty breathing at the time of our visit. Women were noted to have exposure to second-hand smoke if there were any smokers in the household. Physical activity was assessed by obtaining information on the number of hours per day and number of days per week women performed particular tasks (cut and carried wood, ground corn, washed clothes, milked cows, worked in the field, carried heavy items or children, walked normally, walked uphill, and sat relaxing). The number of hours dedicated to each activity was multiplied by a corresponding metabolic equivalent (MET) and summed over a week to evaluate overall physical effort [37].

### 2.6. Statistical Analysis

Data were analyzed using SAS^®^ software version 9.4 (SAS Institute, Inc., Cary, NC, USA). We used descriptive statistics, such as frequencies and relative frequencies, to summarize the exposure measures, health measures, and covariates for all participants who had a FeNO value. We calculated Wilcoxon rank sum tests and Chi-square tests to evaluate statistical differences of covariates between our traditional and cleaner-burning *Justa* stove users.

We calculated Spearman correlation coefficients for the kitchen and personal PM_2.5_ and kitchen and personal black carbon. We used linear regression to assess the association between age and FeNO and height and FeNO. In addition, we evaluated the association between several self-reported health symptoms (cough, chest tightness, current mucus, and difficulty breathing) and FeNO in separate linear regression models adjusting for a-priori confounders age, height, waist-to-hip ratio, BMI, dietary-diversity score, number of assets owned, and years of education.

We used separate linear regression models to evaluate the association between stove type and FeNO, and between each of the four measured pollutants (kitchen PM_2.5_, personal PM_2.5_, kitchen black carbon, and personal black carbon) and FeNO. FeNO and pollutant measurements were natural log transformed to meet model assumptions of residual normality. We chose potential confounders based on a-priori knowledge [38,39]. These included age, height, two measures of obesity (BMI and waist-to-hip ratio), and years of education (less than 6 years or 6 or more years) number of assets, and dietary-diversity score). We evaluated the crude association between FeNO and the various options for confounding by obesity and socio-economic status. Among the obesity and socio-economic status (SES) options, the variable with the strongest crude association with FeNO was chosen for inclusion in the model. All final models were adjusted for age, height, waist-to-hip ratio, and dietary diversity score [40].

We also assessed additive interaction FeNO and age utilizing a dichotomous age variable (less than 40 years and older or equal 40 years old) by including a multiplication term of age and exposure in the both crude and adjusted models [41]. We evaluated the significance of the interaction term with *p*-value of 0.1. We also conducted the analysis after removing five participants who reported exposure to second-hand smoke and after removing persons who reported having a respiratory symptom at the time of measurement (difficulty breathing, sore throat, mucus, tight chest, or cough). Finally, we removed the upper and lower 5% of FeNO values to assess the sensitivity of the model results to extreme values.

## 3. Results

A total of 150 women completed the study; 139 had FeNO measurements, 98 had complete data for measurements of FeNO, fine particulate matter, and black carbon. Baseline population characteristics are presented in Table 1. The average age of women in the study was 37.1 years (SD: 9.1), average BMI was 25.8 kg/m^2^ (SD: 4.2), and about half the population (*n* = 66) had less than 6 years of education. All covariates were similar between to the two stove type groups with the exception of elevation. (Tables S1 and S2 compare the demographic data of the population included in analysis and those excluded due to missing FeNO or exposure).

Fractional exhaled nitric oxide values ranged from 3.5 ppb to 95 ppb, with a mean of 17.9 ppb (standard deviation [SD]:12.1) and median of 15.0 ppb. Among traditional stove users, mean FeNO was 17.4 ppb (SD: 10.8) and the median was 14.5 ppb, while *Justa* stove users had a mean FeNO of 18.5 ppb (SD: 13.4; *p* = 0.64) and median 16.0 ppb. Of the 11 women who did not complete the FeNO measurement, eight women attempted the measurement but were not successful in maintaining their exhaled breath after more than eight attempts. In addition, three women did not attempt the FeNO measurement due to recent surgery or stroke. The 11 women excluded from the analysis had similar exposures to the rest of the sample population (See Supplementary Table S1).

The four 24-h continuous pollutant measures were strongly correlated. Within pollutants, there was a positive correlation between kitchen concentrations and personal concentrations to PM_2.5_ (0.80) and kitchen and personal black carbon (0.77). PM_2.5_ and black carbon exposures were correlated among kitchen measurements (0.89) and personal measurements (0.78). Twenty-four hour average concentrations of each pollutant are shown in Table 2. As expected, kitchen PM_2.5_ was higher than personal PM_2.5_ with means of 254 µg/m^3^ (SD: 329) and 100 µg/m^3^ (SD: 70) respectively. The same pattern holds for kitchen and personal black carbon. In addition, women who owned traditional stoves were exposed to higher concentrations of each of the two pollutants than women who owned *Justa* stoves (*p*-value ≤ 0.01 for all four exposure concentrations).

We did not observe associations between age or height and FeNO (Table 3). Several self-reported respiratory symptoms were associated with increasing FeNO (Table 3). For example, after adjusting for age, height, waist-to-hip ratio, BMI, number of assets, years of education (<6 or ≥6 years), and dietary-diversity score, women who reported having a cough at the time of the assessment had a 78.8% higher FeNO level than those who did not report a cough (95% CI: 38.8%, 130.2%). Similarly, women who reported having current mucus had a 47.4% higher FeNO level (95% CI: 11.2%, 95.2%) than those who did not report current mucus.

Crude and adjusted linear regression results for household air pollution are presented in Table 4. Given the natural log-transformed dependent variables (FeNO) and the natural log-transformed independent continuous pollution exposure variables, we present the results for continuous exposures as the percent increase or decrease in FeNO for a 25% increase in pollutant exposure. Overall results for the continuous pollution measurements were consistent with a null association. For example, a 25% higher personal PM_2.5_ concentration was associated with a 0.8% higher FeNO level (95% CI: −3.1–4.9. The estimates for categorical stove type are presented as a percent increase in FeNO as compared to the reference group (*Justa* stove). Again, the results were consistent with a null association. In Table 4 we also present a sensitivity analysis removing the top and bottom 5% of FeNO values. The results demonstrate that the adjusted models were somewhat sensitive to the highest and lowest FeNO values, but overall, it is difficult to determine the impact, given the wide confidence intervals.

In our interaction analysis, presented in Table 5, participants who were 40 years or older tended to have a larger percent increase in FeNO in relation to exposure to household air pollution compared to the younger participants, although the evidence for interaction was not strong. The additional sensitivity analyses had similar results (results not presented).

## 4. Discussion

Our study provided a unique opportunity to examine associations between measures of exposure to household air pollution and FeNO, a measure of airway inflammation. We add to the limited body of evidence investigating these associations among adults; in particular, our study is, to our knowledge, the first to examine the association of FeNO and household air pollution among healthy adult women using direct exposure measurements of PM_2.5_ and black carbon.

**Figure 3:**
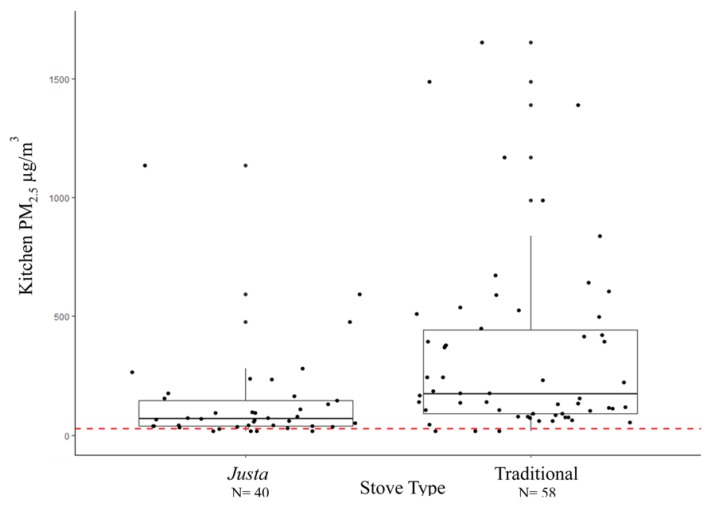
24-h average kitchen PM_2.5_ concentrations, Traditional and *Justa* Stove Users, Rural Honduras (*n* = 98). PM_2.5_: fine particulate matter. Top and bottom lines of rectangle represent the 75th and 25th percentiles. The middle line represents the median. The top whisker denotes the value of the 3rd quartile plus 1.5 times the IQR. The bottom whisker denotes the value of the 1st quartile minus 1.5 times the IQR. The red line indicates the World Health Organization (WHO) 24-h guideline of 25 µg/m^3^.

We observed that although traditional stove users were exposed to an average of 40% higher levels of fine particulate matter compared to *Justa* stove users, the 24-h measures of pollutant exposure for both stove users surpassed the concentration guideline for PM_2.5_ of 25 µg/m^3^ 24-h average set by the World Health Organization (WHO) [42] (Figure 3). It is critical that cleaner-burning cookstove exposure levels are reduced to be as close as possible to the WHO guidelines in order observe population-level improvements on health outcomes. The median FeNO levels observed in our population are slightly higher (14.5 ppb among traditional stove users and 16.0 ppb among *Justa* stove users) than the levels seen in a similar study among a sample of women in Peru using biomass burning cookstoves (10 ppb among traditional stove users and 10.5 ppb among cleaner-burning stove users) [29]. Pollard et al. reported a non-meaningful 2 ppb increase in exhaled nitric oxide among all participants in rural households immediately after a cooking event; however, they did not directly measure pollutant concentrations.

Overall, we did not observe evidence supporting the hypothesis that increased exposure to household air pollution was linked to airway inflammation as measured by FeNO, after adjusting for potential confounders. The evidence from studies on associations of ambient air pollution with FeNO has been inconsistent. For example, although positive associations have been demonstrated between increased levels of ambient PM_2.5_ and black carbon and FeNO among healthy adults [20,43,44], several studies reported null results similar to ours [25,45]. Additionally, several chamber and panel studies of direct exposure to wood smoke and FeNO have also reported results that do not support an association. [46,47,48,49]. There were no differences in the association of measured household air pollution and FeNO among women less than 40 years old and among those 40 years old and older.

FeNO measured at a constant flow rate has been highlighted as a simple, reproducible, non-invasive biomarker of eosinophilic airway inflammation for use in air pollution studies. The inconsistent results observed in the literature may be due in part to the technology available to assess FeNO. Varying the flow rate at which FeNO measurements are collected may allow researchers to partition the source of nitric oxide into two distinct anatomical regions: the proximal and distal airways [50,51]. The current ATS standard is for a flow rate of 50 mL/s, providing information from the proximal airways [14,52,53]. FeNO measured or calculated for a higher flow rate, such as 270 mL/s (FeNO_270_), may be associated with airway inflammation from the distal compartment [51,54].

Previous studies examining the association between air pollution and FeNO at different flow rates have reported inconsistent results for the proximal and distal airways, indicating potential mechanistic differences of exposure [24,49]. For example, a cohort study among 5841 Swedish adults explored the association between ozone and PM_10_ on FeNO measured at 50 mL/s and 270 mL/s, respectively [55]. The authors observed no clear effect of PM_10_ on either measure of FeNO; however, after adjusting for other pollutants, they observed an interquartile range increase in 120-h ozone was associated with a 5.1% (95% CI: 1.7%–8.5%) higher FeNO_270_ measurement and associated (although not significantly) with a 3.6% (−0.4% to 3.4%) higher FeNO_50_ [55]. In addition, a wood smoke chamber study by Barregard et al. 2008 reported a net increase in FeNO_270_ from distal airways three hours post exposure, but no increase in FeNO_50_ from proximal airways [24].

Our study is limited to the approximation of eosinophilic inflammation in the proximal airways measured by a flow rate of 50 mL/s (FeNO_50_). It may be useful for future studies to assess FeNO associated with the distal airways, because PM_2.5_ is small enough to deposit in the distal airways and alveoli [56,57]. Assessing inflammation that may have a stronger expected association to the distal airways would require measurements of exhaled NO at a different flow rate. For example, the semi-portable electrochemical analyser Hypair FeNO mediansoft Exp’air (50, 100, 150 mL/s), could be used in the field setting.

The cross-sectional nature of the study design may limit our ability to establish that exposure preceded airway inflammation. We attempted to address this potential limitation by including only women who had been using a cleaner-burning cookstove for more than four months (average length of *Justa* stove ownership was just under two years). Selection bias could have occurred in our study if those who were enrolled in the study were different—with respect to both exposure and disease—to those who did not participate. For example, if women self-selected into the study because they were exposed to high levels of household air pollution and had poor health, our study results may over exaggerate the association of household air pollution and FeNO. We do not suspect selection bias played a large role in our study, as women were unaware of our health outcome, FeNO; therefore, our population is likely to be similar to the target population with respect to disease.

Potential exposure measurement error is likely to be non-differential with respect to measured FeNO values. Likewise, disease measurement error is likely non-differential with respect to measured exposure. Any measurement error of the exposure could have resulted in a bias towards the null, while measurement error in our continuous outcome would not result in bias. The results are limited, and may not be generalizable to other populations, especially those outside Central America, or to populations using different stove and fuel combinations.

Although we had a relatively small sample size, the association between FeNO and self-reported respiratory symptoms indicates that we may have accurately captured airway inflammation as it relates to the presence of symptoms [39,58]. While we did not see an association between FeNO and age or FeNO and height, as we would have expected based on previous literature, [38,39,58,59,60], this may be due to the relatively small range of age and height in our study. For example, our study population only covered adults age 25–56 (mean 37.1 years, SD: 9.1 years). In addition, we observed very little variability in height (mean: 1.45 m, SD: 0.05 m), which may have influenced our inability to capture an association between FeNO and height. Our crude and adjusted linear regression models were not substantially different, indicating that measured confounding was likely not a concern in our study; however, measurement error or residual confounding, for example by SES, may still exist. FeNO can be impacted by diet, particularly green, leafy vegetable consumption [61]. Although a dietary recall survey conducted in our study population was not specific enough to capture this intake, overall report of vegetable consumption was low (median intake, 0 servings). Finally, we did not include questions in our demographic and health survey that would provide insight into the asthma, atopy, or genetic variations among participants in order to assess effect modification. No participants, however, reported taking any asthma medication. Asthma or atopy status and genetic variations are thought to modify the effect of air pollution on FeNO [56,62,63]; similar effect modification could be expected in household air pollution. We did not evaluate former smoking among our study population, and cannot determine if former smoking may contribute to the relatively low FeNO levels observed in our study. Smoking however, is uncommon among the rural female population in Honduras.

## 5. Conclusions

In conclusion, we did not observe evidence of increased eosinophilic airway inflammation from exposure to household air pollution. Further research is needed to assess the utility of FeNO in the field of household air pollution. Future studies may find it useful to consider measurements of this biomarker at differing airflow rates, in well-designed longitudinal studies.

## Figures and Tables

**Table 1:** Population characteristics among nonsmoking primary female cooks using traditional or cleaner-burning *Justa* stoves, rural Honduras (*n* = 139).

	Total (*n* = 139)	Traditional (*n* = 72)	*Justa* (*n* = 67)	Test Value
	*n* (%) or Mean (SD); Range	*n* (%) or Mean (SD); Range	*n* (%) or Mean (SD); Range	(*p*-Value) *
Age (years)	37.1 (9.1); 25–56	38.3 (9.9); 25–56	35.9 (7.9); 25–56	0.29
Height (meters)	1.45 (0.05); 1.37–1.59	1.45 (0.05); 1.37–1.59	1.45 (0.04); 1.37–1.56	0.40
Waist-to-hip ratio	0.87 (0.06); 0.74–1.10	0.88 (0.06); 0.74–1.09	0.87 (0.05); 0.77–0.99	0.08
Body mass index (kg/m^2^)	25.8 (4.2); 17.6–37.5	25.5 (4.4); 17.5–37.5	26.2 (3.8); 18.2–33.6	0.24
Physical activity (MET) ^1^	212 (103); 31–542	216 (110); 31–542	209 (95); 46–444	0.82
Elevation (meters)	1916 (107); 1729–2157	1896 (98); 1736–2152	1938 (112); 1729–2157	0.01
Beds per person ^2^	0.52 (0.18); 0.2–1.0	0.50 (0.17); 0.2–1.0	0.55 (0.19); 0.25–1.0	0.15
Diet diversity score ^3^	6.1 (1.7); 2–10	6.1 (1.7); 3–10	5.9 (1.6); 2–10	0.62
Years of education				
Less than six years	66 (48.1%)	38 (53.5%)	28 (42.4%)	0.26
Six or more years	71 (51.8%)	33 (46.5%)	38 (57.6%)	
Number of assets ^4^				
Less than two	67 (48.5%)	38 (53.5%)	34 (51.0%)	0.87
Two or more	71 (51.5%)	33 (46.5%)	33 (49.0%)	
Years spent cooking with biomass	25.6 (9.9); 7–50	26.6 (10.8); 7–49	24.5 (8.8); 9–50	0.38
Self-reported exposure to secondhand smoke	5 (3.6%)	5 (3.6%)	0 (0%)	-
Fractional exhale nitric oxide (ppb)	17.9 (12.1); 3.5–95	17.4 (10.8); 3.5–62	18.5 (13.4); 5–95	0.64

PPB, parts per billion; SD, Standard Deviation. ^1^ Physical Activity: The sum of metabolic equivalents including the following self-reported activities: cut wood, grind corn, wash clothes, milk the cow, work in the field, carry a heavy weight, and walk normally outside the house. For each activity the number of hours per week was calculated and multiplied with the corresponding metabolic equivalent (MET) from the Compendium of Physical Activities (Ainsworth et al. 2015). ^2^ Total *n* = 137; Traditional = 71; *Justa* = 66. ^3^ Dietary-diversity score: The sum of the number of food categories consumed in the past 24-h (10 categories); used as an indicator of socioeconomic status (Savy et al. 2006). ^4^ Number of assets (Total *n* = 138, Traditional = 71). Assets include cars, bikes, motorbikes, televisions, radios, refrigerators, sewing machines, electricity. * Wilcoxon rank sum test for continuous variables or chi-square *p*-value for categorical variables.

**Table 2:** 24-h average kitchen and personal fine particulate matter and black carbon concentrations, traditional and *Justa* stove users, rural Honduras. PM_2.5_: fine particulate matter.

	All Participants						Traditional Stove Users						*Justa* Stove Users					
	*n*	Min	25th	Median	75th	Max	*n*	Min	25th	Median	75th	Max	*n*	Min	25th	Median	75th	Max
24-h average kitchen PM_2.5_ (µg/m^3^)	98	18	61	116	369	1654	58	18	90	172	448	1654	40	18	37	68	150	1134
24-h average personal PM_2.5_ (µg/m^3^)	98	18	48	80	138	346	59	18	62	112	154	346	39	18	39	52	81	174
24-h average kitchen Black Carbon (µg/m^3^)	98	1	8	18	78	1172	58	1	14	44	113	1172	40	1	4	11	15	469
24-h average personal Black Carbon (µg/m^3^)	98	1	4	7	17	123	58	1	6	14	32	123	40	1	1	4	8	47

**Table 3:** Estimated crude and adjusted percentage difference in fractional exhaled nitric oxide in relation to measures of current, self-reported symptoms among traditional and *Justa* stove users, rural Honduras.

	*n*	Crude Percent Difference in FeNO	95% CI	*n*	Adjusted Percent Difference in FeNO	95% CI
Age (years) ^1^	139	<0.1	(−0.2, 0.3)	136	0.1	(−0.2, 0.4)
Height (meters) ^2^	139	−3.79	(−39.51, 53.02)	136	−0.25	(−37.59, 59.44)
Cough ^3^						
No	118	ref		115		
Yes	21	78.8	(38.8, 130.2)	21	78.8	(37.5, 132.5)
Chest Tightness ^3^						
No	128	ref		125		
Yes	11	17.6	(−17.9, 68.3)	11	24.5	(−14.5, 81.5)
Mucus ^3^						
No	121	ref		118		
Yes	18	47.4	(11.2, 95.3)	18	52.4	(13.4, 104.8)
Difficulty Breathing ^3^						
No	129	ref		126		
Yes	10	42.1	(−2.0, 105.9)	10	39.4	(−5.1, 104.8)

Cl: Confidence interval; PM_2.5_: fine particulate matter. ^1^ Model was adjusted for height, waist-to-hip ratio, body mass index, dietary-diversity score, years of education (<6 or ≥6 years), and number of assets (<2 or ≥2) (Assets include cars, bikes, motorbikes, televisions, radios, refrigerators, sewing machines, electricity). ^2^ Model was adjusted for age, waist-to-hip ratio, body mass index, dietary-diversity score, years of education (<6 or ≥6 years), and number of assets (<2 or ≥2) (Assets include cars, bikes, motorbikes, televisions, radios, refrigerators, sewing machines, electricity). ^3^ Exhaled nitric oxide was log-transformed. Categorical variable beta coefficients were entered into the formula (e^β − 1)*100). The estimates for the categorical measures of exposure can be interpreted as the percent difference in FeNO when comparing those who had the health system to those who did not.

**Table 4:** Estimated crude and adjusted ^1^ percentage difference in fractional exhaled nitric oxide in relation to measures of exposure to household air pollution (per 25% increase in 24-h average measured pollution, or by stove type) among traditional and *Justa* stove users, rural Honduras.

	*n*	Crude Percent Difference in FeNO	95% CI	*n*	Adjusted Percent Difference in FeNO ^1^	95% CI
24-h average kitchen PM_2.5_ (µg/m^3^) ^2^	98	0.3	(−2.0, 2.7)	84	0.5	(−2.0, 3.1)
24-h average personal PM_2.5_ (µg/m^3^) ^2^	98	0.8	(−3.1, 4.9)	85	0.8	(−3.4, 5.2)
24-h average kitchen Black Carbon (µg/m^3^) ^2^	98	−0.1	(−1.8, 1.6)	84	−0.1	(−1.9, 1.8)
24-h average personal Black Carbon (µg/m^3^) ^2^	98	<0.0	(−2.1, 1.9)	84	−0.2	(−2.4, 2.0)
Stove Type ^3^	139			136		
*Justa*	67	ref		65	ref	
Traditional	72	−6.5	(−22.9, 13.6)	71	−6.1	(−23.5, 15.3)

Cl: Confidence interval; PM_2.5_: fine particulate matter. ^1^ Models were adjusted for age, height, waist-to-hip ratio, body mass index, dietary-diversity score, years of education (<6 or ≥6 years), and number of assets (<2 or ≥2) (Assets include cars, bikes, motorbikes, televisions, radios, refrigerators, sewing machines, electricity). ^2^ Exhaled nitric oxide and measured pollution were both log transformed. Beta coefficients were entered into the formula ((1.25^β) − 1) and multiplied by 100. We can interpret the estimate of the continuous pollution exposures as a percent increase in exhaled nitric oxide for each 25% increase in exposure. Example: There is a 0.4% higher FeNO level with a 25% higher kitchen PM_2.5_ concentration. ^3^ Exhaled nitric oxide was log-transformed. Categorical variable beta coefficients were entered into the formula (e^β − 1)*100). The estimates for the categorical measures of exposure can be interpreted as the percent difference in FeNO when comparing traditional stove to the reference (*Justa* stove).

**Table 5:** Estimates for effect modification by age (dichotomized at the median value of 40 years) for the percentage difference in fractional exhaled nitric oxide in relation to measures of exposure to household air pollution (per 25% increase in 24-h average measured pollution, or by stove type) among traditional and *Justa* stove users, rural Honduras ^1^.

	*n*	Adjusted Percent Difference ^1^	95% CI	*p*-Value for Interaction
24-h average kitchen PM_2.5_ (µg/m^3^) ^2^				0.7
Age < 40	65	<0.1	(−3.3, 3.4)	
Age ≥ 40	31	1.0	(−3.0, 5.1)	
24-h average personal PM_2.5_ (µg/m^3^) ^2^				0.2
Age < 40	66	−0.9	(−5.7, 4.3)	
Age ≥ 40	31	5.2	(−3.3, 14.3)	
24-h average kitchen Black Carbon (µg/m^3^) ^2^				0.8
Age < 40	65	−0.3	(−2.6, 2.1)	
Age ≥ 40	31	0.2	(−2.7, 3.2)	
24-h average personal Black Carbon (µg/m^3^) ^2^				0.7
Age < 40	65	−0.5	(−3.1, 2.3)	
Age ≥ 40	31	0.5	(−3.3, 4.7)	
Stove Type ^3^ (traditional compared to *Justa* [ref])				
Traditional				
Age < 40	72	−12.2	(−31.9, 13.1)	0.3
Age ≥ 40	67	11.1	(−22., 59.2)	

Cl: Confidence interval; PM_2.5_: fine particulate matter. ^1^ All models adjusted for age, height, waist-to-hip ratio, body mass index, dietary-diversity score, years of education (<6 or ≥6 years), and number of assets (<2 or ≥2) (Assets include cars, bikes, motorbikes, televisions, radios, refrigerators, sewing machines, electricity). ^2^ Exhaled nitric oxide and measured pollution are both log transformed. Beta coefficients were entered into the formula ((1.25^β) − 1) and multiplied by 100. We can interpret the estimate of the continuous pollution exposures as a percent increase in exhaled nitric oxide for each 25% increase in exposure. Example: There is a 0.2% higher FeNO level with a 25% higher kitchen PM_2.5_ concentration among women less than 40 years old. ^3^ Exhaled nitric oxide was log-transformed. Categorical variable beta coefficients were entered into the formula (e^β − 1)*100). The estimates for the categorical measures of exposure can be interpreted as the percent difference in FeNO when comparing a specific stove type to the reference (traditional stove).

## Supplementary Materials

The following are available online at http://www.mdpi.com/1660-4601/15/11/2544/s1, Table S1: Population characteristics of nonsmoking primary female cooks in rural Honduras included in the study and participants excluded due to missing FeNO data, Table S2: Population characteristics of nonsmoking primary female cooks in rural Honduras included in the study and participants excluded due to missing exposure data (PM_2.5_), Table S3: Estimated crude and adjusted percentage difference in fractional exhaled nitric oxide in relation to measures of exposure to household air pollution (per 25% increase in 24-h average measured pollution, or by stove type) among traditional and *Justa* stove users, rural Honduras with (Top and Bottom 5% removed).
