# Supplementary material for: Exposure to Household Air Pollution from Biomass Cookstoves and Levels of Fractional Exhaled Nitric Oxide (FeNO) among Honduran Women
Source: Int J Environ Res Public Health. 2018 Nov 13;15(11):2544. doi: 10.3390/ijerph15112544 (PMC6267103; doi:10.3390/ijerph15112544)
Supplement: Supplementary file 1 [file ijerph-15-02544-s001.pdf]

S1: Population characteristics of nonsmoking primary female cooks in rural Honduras included in the study and participants excluded due to missing FeNO data

	Total (N=139) N (%) or Mean (SD) ; range	No FeNO (N=11) N (%) or Mean (SD) ; range	Test value (p-value)*
Age (years)	37.1 (9.1); 25-56	39.6 (7.6); 27-48	0.28
Height (inches)	57.0 (1.8); 53.8-62.5	55.6 (1.7); 53.8-59.0	0.01
Waist-to-hip ratio	0.87 (0.06); 0.7-1.1	0.88 (0.06); 0.8-1.0	0.79
Body mass index (kg/m^2)	25.8 (4.2); 17.6-37.5	24.8 (5.1); 17.1-36.5	-0.27
Physical activity (MET)[1]	212 (103); 31-542	201 (147); 42-491	0.50
Elevation (meters)	1916 (107); 1729-2157	1910 (110); 1760-2171	0.92
Beds per person[2]	0.52 (0.18); 0.2-1.0	0.51 (0.18); 0.25-0.8	0.91
Diet diversity score[3]	6.1 (1.7); 2-10	5.8 (1.4); 4-8	0.57
Years of education			
Less than six years	66 (48.1%)	4 (36.4%)	0.67
Six or more years	71 (51.8%)	7 (53.6%)	
Number of assets[4]			
Less than two	67	4	0.65
Two or more	71	7	
Years spent cooking with biomass	25.6 (9.9); 7-50	28.4 (8.2); 15-37	0.32
Self-reported exposure to secondhand smoke	5 (3.6%)	0 (0%)	-
Fractional exhale nitric oxide (ppb)	17.9 (12.1); 3.5-95	-	-

PPB, parts per billion; SD, Standard Deviation

[1]Physical Activity: The sum of metabolic equivalents including the following self-reported activities: cut wood, grind corn, wash clothes, milk the cow, work in the field, carry a heavy weight, and walk normally outside the house. For each activity the number of hours per week was calculated and multiplied with the corresponding metabolic equivalent (MET) from the Compendium of Physical Activities (Ainsworth et al. 2015).

[2] Total N=137; Traditional = 71; Justa = 66

[3] Dietary-diversity score: The sum of the number of food categories consumed in the past 24-hours (10 categories); used as an indicator of socioeconomic status (Savy et al. 2006)

[4]Number of assets (Total N=138, Traditional=71). Assets include cars, bikes, motorbikes, televisions, radios, refrigerators, sewing machines, electricity.

*Wilcoxon rank sum test

S2: Population characteristics of nonsmoking primary female cooks in rural Honduras included in the study and participants excluded due to missing exposure data ($PM_{2.5}$)

	PM (N=98) N (%) or Mean (SD) ; range	No PM (N=41) N (%) or Mean (SD) ; range	Test value (p-value)*
Age (years)	36.8 (9.1); 25-56	37.8 (9.2); 25-56	0.55
Height (meters)	1.44 (0.04); 1.37-1.55	1.46 (0.5); 1.37-1.59	0.17
Waist-to-hip ratio	0.88 (0.06); 0.74-1.10	0.88 (0.1); 0.77-0.99	0.55
Body mass index (kg/m2)	26.4 (4.0); 18.4-37.5	24.6 (4.2); 17.6-32.7	0.04
Physical activity (MET)1	217 (113); 31-543	202 (75); 100-394	0.70
Elevation (meters)	1878 (71); 1729-2105	2001 (122); 1827-2157	<0.01
Beds per person2	0.53 (0.18); 0.3-1.0	0.52 (0.2); 0.23-1.0	0.74
Diet diversity score3	6.0 (1.7); 3-10	6.4 (1.7); 2.0-10.0	0.16
Years of education			
Less than six years	42 (43.7%)	24(58.5%)	0.13
Six or more years	54 (56.3%)	17 (41.5%)	
Number of assets4			
Less than two	41 (41.8%)	27 (65.8%)	0.02
Two or more	57 (58.2%)	14 (34.2%)	
Years spent cooking with biomass	25.3 (10.0); 7-49	26.2 (10.0); 12.0-50.0	0.84
Self-reported exposure to secondhand smoke	4 (4.1%)	1 (2.4%)	-
Fractional exhale nitric oxide (ppb)5	18.8 (13.0); 3.5-95	15.9 (9.4); 5.0-45.0	0.23

PPB, parts per billion; SD, Standard Deviation

[1]Physical Activity: The sum of metabolic equivalents including the following self-reported activities: cut wood, grind corn, wash clothes, milk the cow, work in the field, carry a heavy weight, and walk normally outside the house. For each activity the number of hours per week was calculated and multiplied with the corresponding metabolic equivalent (MET) from the Compendium of Physical Activities (Ainsworth et al. 2015).

[2] Those without PM N=40 (Traditional=26; Justa=14)

[3] Dietary-diversity score: The sum of the number of food categories consumed in the past 24-hours (10 categories); used as an indicator of socioeconomic status (Savy et al. 2006)

[4]Number of assets (People with PM, N=96). Assets include cars, bikes, motorbikes, televisions, radios, refrigerators, sewing machines, electricity.

*Wilcoxon rank sum test or chi-square test for categorical variables

S3: Estimated crude and adjusted[1] percentage difference in fractional exhaled nitric oxide in relation to measures of exposure to household air pollution (per 25% increase in 24-hour average measured pollution, or by stove type) among traditional and *Justa* stove users, rural Honduras with (Top and Bottom 5% removed)

Top and Bottom 5% Removed	N	Crude Estimate	95% CI	N	Adjusted Estimate*	95% CI
24–hour average kitchen $PM_{2.5}$ (μg/m3)[2]	86	0.3	(-2.0, 2.7)	84	-0.01	(-2.2, 2.2)
24–hour average personal $PM_{2.5}$ (μg/m3)[2]	86	0.8	(-3.1, 4.8)	85	-0.01	(-3.5, 3.7)
24–hour average kitchen Black Carbon (μg/m3)[2]	86	0.4	(-1.0, 1.9)	84	0.10	(-1.5, 1.7)
24–hour average personal Black Carbon (μg/m3)[2]	86	0.2	(-1.5, 1.9)	85	-0.29	(-2.2, 1.6)
Stove Type[3]	122			119		
Justa	59	ref		57		
Traditional	63	5.3	(-10.6, 24.0)	62	3.8	(-12.5, 23.1)

Cl: Confidence interval; PM2.5: fine particulate matter

*Adjusted N (Kitchen PM2.5 = 84, personal PM2.5 =85, kitchen black carbon = 84, personal black carbon =85)

1Models were adjusted for age, height, waist-to-hip ratio, body mass index (BMI), dietary-diversity score, years of education (<6 or ≥6 years), and number of assets (<2 or ≥2) (Assets include cars, bikes, motorbikes, televisions, radios, refrigerators, sewing machines, electricity).

2Exhaled nitric oxide and measured pollution were both log transformed. Beta coefficients were entered into the formula ((1.25^β)-1) and multiplied by 100. We can interpret the estimate of the continuous pollution exposures as a percent increase in exhaled nitric oxide for each 25% increase in exposure. Example: There is a 0.4% higher FeNO level with a 25% higher kitchen PM2.5 concentration.

3Exhaled nitric oxide was log-transformed. Categorical variable beta coefficients were entered into the formula (e^β-1)*100). The estimates for the categorical measures of exposure can be interpreted as the percent difference in FeNO when comparing traditional stove to the reference (Justa stove).

S4 Methods: Black Carbon Estimation

We estimated $PM_{2.5}$ black carbon concentrations based on the optical transmission of light through the air sampling filters. A transmissometer (model OT-21, Magee Scientific, USA) estimated the attenuation at 880 nm light intensity through the sample filter, which is proportional to the amount of black carbon on the filter. To estimate the black carbon loading we first define a measure of attenuation (ATN) as the natural log of the ratio of light transmittance of a reference filter (I_0) to a sample filter (I) multiplied by 100:

$$ATN = 100 \times ln\left(\frac{I_0}{I}\right) \quad (1)$$

We used a single value for reference transmittance (I_0 = 224571), taken as the average transmittance of 54 field blank filters. This reference method is similar to that reported previously with laboratory blank filters (Presler-Jer et al) and one that also allows us to account for contamination that may have occurred with filter handling during non-sampling periods.

The measured attenuation was then used to derive the attenuation coefficient (b_{atn}) in units of inverse megameters (Mm^{-1}), adjusting for field sampling factors such as the sampled area on the filter (m^2), and the volume of the air sampled (m^3, calculated using the sample flow rate and the sample duration). The attenuation coefficient was calculated as described by Presler-Jur et al:

$$b_{atn} = \frac{Filter\ Area}{Sample\ Volume} \times ATN \times 10^4 \quad (2)$$

Assumptions of black carbon concentration estimates have uncertainties given the properties of particles (e.g. differences in light scattering and combustion source). We used a mass attenuation cross-section), σ_{atn}, to convert from ATN to an equivalent BC concentration, which implies a linear relationship between the BC and the ATN of the sample filter. To account for the primarily wood-burning nature of the exposure, we defined σ_{atn} = 12.5 m^2/g as derived previously for carbonaceous smoke by Chylek et al., 1981. Additionally, previous studies have demonstrated a measurement artifact wherein an underestimation of the ATN becomes more pronounced at higher black carbon concentration. We therefore used a loading correction r, calculated according Kirchstetter and Novakov 2007:

$$r = \left(exp^{-ATN/100}\right) \times 0.88 + 0.12 \quad (3)$$

The final estimated BC concentration (BC, $\mu g/m^3$) was calculated as follows:

$$BC = \frac{b_{atn}}{\sigma_{atn} \times r} \quad (4)$$

We used a single value for reference transmittance (I0 = 224571), taken as the average transmittance of 54 field blank filters. This reference method is similar to that reported previously with laboratory blank filters (Presler-Jer et al) and one that also allows us to account for contamination that may have occurred with filter handling during non-sampling periods. Although these field blanks were not collected during the same sampling period; samples were collected within a year and with similar field methods.
